# Assessment of Health-Promoting Lifestyle Profile: A Cross-Sectional Study of Adolescents and Young Adults in Mongolia

**DOI:** 10.3390/ijerph22101485

**Published:** 2025-09-25

**Authors:** Erdenezul Uitumen, Klára Tarkó

**Affiliations:** 1 Doctoral School of Education, Faculty of Humanities and Social Sciences, University of Szeged, 6722 Szeged, Hungary; 2 MTA-SZTE Health Promotion Research Group, 6725 Szeged, Hungary; 3 Department of Nutrition, School of Public Health, Mongolian National University of Medical Sciences, Ulaanbaatar 14210, Mongolia; 4 Institute of Applied Health Sciences and Environmental Education, Juhász Gyula Faculty of Education, University of Szeged, 6725 Szeged, Hungary

**Keywords:** health-promoting lifestyle, health education, adolescent, young adult, university

## Abstract

The study examined health-promoting lifestyles and their contributing factors among adolescents and young adults at three universities in Ulaanbaatar, Mongolia. A cross-sectional study was conducted from April to May 2024. A total of 827 participants were analyzed using the Health-Promoting Lifestyle Profile II (HPLP-II). Exploratory and confirmatory factor analyses were performed using JASP to ensure cultural adaptation. Descriptive statistics, an independent *t*-test, one-way ANOVA, and a MANOVA were employed to analyze the data in SPSS. Post hoc analyses and effect sizes were conducted in cases of significance. The mean HPLP-II score was 2.60 (SD = 0.35). The highest mean score was on the interpersonal relationships subscale (M = 2.89; SD = 0.52), and the lowest was on the health responsibility subscale (M = 2.31; SD = 0.49). Our findings revealed that there were significant differences in the overall HPLP-II scores based on university, working status, and economic status. Additionally, the data revealed that for certain HPLP-II subscales, factors such as gender, residence type, and location of graduation schools were significant. Universities are potential settings for planning and implementing health promotion programs that encourage adolescents and young adults to take responsibility for their health and engage in physical activity.

## 1. Introduction

Non-communicable diseases (NCDs), also referred to as chronic diseases, have long-term consequences and result from unhealthy behavioral, genetic, physiological, and environmental factors. The most widely prevalent forms of NCDs are cancer, cardiovascular disease, and diabetes. The World Health Organization (WHO) [1] reported that people of all ages are at risk of NCDs due to physical inactivity, unhealthy diets, alcohol consumption, and smoking. In addition, these behaviors are the leading causes of high blood pressure, overweight and obesity, high blood glucose, and high blood lipids.

The transition to university represents a pivotal point in the lives of adolescents and young adults (AYAs) characterized by the pursuit of autonomy from their parents, the emergence of behavioral changes, and changes in their social and physical environment [2,3]. Studies have confirmed that university students are prone to engaging in health-damaging behaviors, such as unhealthy eating, a lack of physical activity, and poor sleep and rest, which can further affect long-term health [4].

The Ottawa Charter for Health Promotion defined health promotion as “the process of enabling people to increase control over, and to improve their health” [5]. Later, the Bangkok Charter [6] updated the Ottawa Charter, addressing health promotion from an individual healthy lifestyle education model to a socio-ecological model that determines social determinants of health. The document emphasized the health promotion theory and practice, considering the roles of social and personal resources, physical ability, and the importance of achieving health equity. The term “lifestyle” is widely understood to encompass discretionary activities that are routinely integrated into an individual’s daily living patterns and play a crucial role in shaping overall health status. A health-promoting lifestyle is characterized by the inherent human drive toward self-actualization, which contributes to enhanced well-being, personal fulfillment, and a more productive life [7].

In Mongolia, there is a paucity of research that has demonstrated that adolescents and young adults are vulnerable to health-risk behaviors, including alcohol and tobacco use, poor nutrition, risky sexual behaviors, and mental health problems [8,9,10,11,12]. Since 1990, the Mongolian government has been implementing measures to improve the quality of life, build commitment, and reorganize policies and regulations to prevent and control NCDs in accordance with the WHO action plans. However, the implications are challenging, as studies have shown that the Mongolian population is at significantly high risk of increasing NCDs due to alcohol consumption and smoking, overweight, physical inactivity, poor diet, and low levels of healthcare seeking [13,14,15].

The Health-Promoting Lifestyle Profile II (HPLP-II) was developed and introduced by Walker et al. [16] based on Pender’s Health Promoting Model (HPM) [7]. The model is a pattern for demonstrating health-promoting behaviors, encouraging people to focus on increasing their level of well-being, self-actualization, and personal fulfillment. Also, Pender et al. [7] demonstrated that personal factors for individuals may influence their health-promoting behaviors. These sociodemographic factors are classified into biological, psychological, and sociocultural factors. For instance, biological factors considered in this analysis included age, body mass index (BMI), and physical strength or balance. Psychological factors may include self-confidence, motivation, and health perception. Whereas sociocultural factors include race, ethnicity, education, and socioeconomic background. The HPLP-II instrument is a widely used and cross-culturally validated tool employed to examine lifestyle behavior in university settings [17,18,19,20,21,22,23]. The assessment of health-related lifestyles is essential for the provision of information for health promotion among adolescents and young adults [1]. A number of international studies have reported associations between sociodemographic factors and health-promoting lifestyles [24,25,26,27,28,29,30,31,32,33,34,35,36,37,38,39,40]. Consequently, there is a necessity to conduct further research on the health-promoting lifestyles among Mongolian university students to develop suitable health interventions aimed at enhancing their health and well-being.

The objective of the present study was firstly to identify the present status of health-promoting lifestyle and secondly to explore any differences along sociodemographic factors in the HPLP-II scores among AYAs in Ulaanbaatar city, Mongolia. To our knowledge, this is the first study to investigate the health-promoting lifestyle of AYAs using the HPLP-II in a Mongolian context. Therefore, the objectives of the study were as follows:

1. To determine if there are any significant differences in the HPLP-II and subscales scores across sociodemographic factors, such as gender, academic year, BMI, place of residence, cigarette use, and financial status.
2. To explore the differences between the HPLP-II score and AYAs’ level of health education, as well as their opinions of the “Health subject” taught in secondary school.

## 2. Materials and Methods

### 2.1. Participants and Procedure

A cross-sectional study design was obtained from April to May of 2024. A total of 900 questionnaires were distributed, and 827 were completed in an appropriate manner, representing a response rate of 92.0%. AYAs were university students (18–24 years), categorized into late adolescence (18–20 years) and young adulthood (21–24 years) to capture the critical developmental transitions [41].

The data were collected from three of the largest public universities between April and May 2024, in Ulaanbaatar City, Mongolia: the National University of Mongolia (NUM), the Mongolian State University of Education (MSUE), and the Mongolian University of Science and Technology (MUST). Prior to data collection, the departments were contacted, and the objective of the study was explained, and asked to assist in the distribution of the questionnaires. A convenience sampling method was employed to recruit the participants. The administration of the questionnaires to the participants occurred in the classroom, and the questionnaires were completed on paper forms. The questionnaires were anonymous. After completing the questionnaires, the students returned to the instructors. No incentives were provided to the respondents. Data were collected from a variety of academic disciplines, including education, the humanities and the social sciences, and engineering.

The study was conducted after obtaining ethical approval from the Institutional Review Board of the Doctoral School of Education, University of Szeged (date: 10 April 2024) with reference number 9/2024. The AYA participants were asked to participate in the study at the end of their class and were informed of the purpose of the study. Additionally, the respondents were informed at the beginning of the questionnaire that their participation was voluntary and responses would be kept confidential. The participants indicated their willingness to participate in the study by selecting the “I agree” response, and all procedures were conducted in accordance with the Declaration of Helsinki.

### 2.2. Measures

In this study, the AYAs were asked to complete a self-administered questionnaire comprising two sections. The first section of the survey contained items pertaining sociodemographic characteristics, including age, gender, academic year, cigarette use, body mass index (BMI: <18.5—underweight, 18.5–24.9—normal, and >25.0—overweight), location of graduated secondary school (Ulaanbaatar city and rural areas), type of residence (living with parents, dormitory, and other), economic status (good, average, and less than average), and whether the students were employed part-time. In addition, the first section of the survey included items regarding the participants’ experiences with health education (good, average, and poor) as well as their opinion of the “Health subject” taught in secondary school (good, average, and poor).

The second section of the questionnaire focused on AYAs’ health-promoting lifestyles and practices using the HPLP-II developed by Walker et al. [16]. The tool consists of 51 items with six subscales: health responsibility (8 items), nutrition (9 items), physical activity (8 items), spiritual growth (9 items), interpersonal relationships (9 items), and stress management (8 items). The scale utilizes a four-point Likert scale to assess the frequency of each behavior, ranging from 1—never, 2—sometimes, 3—often, to 4—always. A mean score of ≥2.50 for the respondents was considered a positive response, which is consistent with the findings of previous studies [26,28].

The HPLP-II was translated into the Mongolian language following the standard forward and backward translation procedure [42]. A pilot study was conducted among 376 Mongolian university students to ensure the cultural and linguistic appropriateness of the translated version of the questionnaire. The students encountered no significant challenges in completing the questionnaire. The items were all clear and easily comprehensible. The overall Cronbach’s α for the Mongolian HPLP-II in this study was 0.90, and for the subscales, it ranged between 0.52 and 0.84. The nutrition subscale demonstrated the lowest internal consistency (Cronbach’s α = 0.52). Nevertheless, given the satisfactory overall scale reliability, the nutrition subscale items were retained. However, it is imperative that subsequent studies consider revising these items to improve reliability.

To ensure the cultural adaptation of the HPLP-II within the Mongolian context, we conducted exploratory factor analysis (EFA) and confirmatory factor analysis (CFA) to confirm the validity of the questionnaire’s structure. The EFA was conducted to confirm the variance of the HPLP-II questionnaire. The coefficient of Kaiser–Meyer–Olkin for 51 items was 0.88. The significance of Bartlett’s Test Sphericity was 0.00 (χ^2^ = 6640.71, df = 1326, *p* < 0.001), indicating that samples met the criteria for the factor analysis. The CFA measurement with 51 items indicated a good fit to the data based on χ^2^ = 3818.76, df = 1160, *p* < 0.001, Comparative Fit Indices (CFI = 0.95), Tucker and Lewis Index (TLI = 0.94), Goodness of Fit Indices (GFI = 0.93), and Root Mean Square Error of Approximation (RMSEA = 0.042).

### 2.3. Data Analysis

The data were analyzed using the Statistical Package SPSS version 25 (SPSS) (IBM Corp., Armonk, NY, USA). Descriptive statistics were used for the sociodemographic variables, such as frequencies, means, and standard deviations. Independent *t*-test and one-way ANOVA were used to determine and compare differences in the HPLP-II scores according to sociodemographic factors. For any statistically significant ANOVA result, post hoc comparisons were conducted using Tukey’s HSD analysis. The magnitude of the significant differences was calculated as effect sizes using Cohen’s d for the *t*-test. Lastly, we conducted multivariate analysis of variance (MANOVA) to examine differences between gender and economic status on HPLP-II scores. Two-tailed significance was considered at *p* < 0.05.

## 3. Results

### 3.1. Participants

A total of 827 AYA respondents completed the questionnaires appropriately, of whom 251 (30.4%) were enrolled at the MSUE, 277 (33.5%) at the NUM, and 299 (36.2%) at the MUST. The sample included 330 (39.9%) male and 497 (60.1%) female participants. The mean age of the respondents was 19.4 (SD = 1.37) years, ranging from 18 to 25 years. Of the participants, 352 (42.6%) completed secondary education in Ulaanbaatar city, while 475 (57.4%) were from rural areas of Mongolia. In total, 358 (46.3%) AYAs lived with their parents, while 168 (20.3%) resided in dormitories, 153 (18.5%) were renting apartments, and 123 (14.9%) lived in other types of housing. The majority of the participants (83.1%) were non-smokers. According to the experiences of health education, 182 (22.0%) of the participants considered it as “good,” 661 (73.9%) as “average,” and 34 (4.1%) as “poor.” In addition, the participants expressed their opinions on the subject of Health education taught in their secondary schools. The majority of the AYAs (*n* = 554, 67.0%) responded “average,” while 109 (13.2%) of them considered it as “good,” and 160 (19.3%) rated it as “poor.” Table 1 shows the sociodemographic and healthy lifestyle-related characteristics of the participants.

### 3.2. Gender Differences

The overall mean score for the HPLP-II was 2.60 (SD = 0.35). The interpersonal relationship subscale had the highest mean score (M = 2.89; SD = 0.52), and the health responsibility subscale had the lowest mean score (M = 2.31; SD = 0.49). In terms of gender differences, male participants had an overall mean score of 2.59 (SD = 0.35) on the HPLP-II, while female participants had an overall mean score of M = 2.61 (SD = 0.35). There was no significant difference between male and female AYA participants in the total HPLP-II score. However, the difference in the health responsibility scores between female (M = 2.35, SD = 0.47) and male (M = 2.24, SD = 0.50) participants was statistically significant, with a small effect size (Cohen’s d = 0.25). The difference in nutrition scores between females (M = 2.70, SD = 0.33) and males (M = 2.65, SD = 0.40) was statistically significant, with small effect size (Cohen’s *d* = 0.13). The difference in physical activity scores between female (M = 2.23, SD = 0.63) and male (M = 2.47, SD = 0.61) participants was statistically significant, with a small effect size (Cohen’s d = 0.38). The difference in interpersonal relationships scores between female (M = 2.96, SD = 0.51) and male (M = 2.77, SD = 0.52) participants was statistically significant, with a small effect size (Cohen’s d = 0.37) (see Table 2).

### 3.3. Type of Residence

There were no significant differences in the overall HPLP-II scores among AYAs according to residence type. However, AYAs living with their families had significantly higher scores on the nutrition subscale. Post hoc analyses using Tukey’s HSD test were conducted to analyze the significant effect. The results indicated that the participants living with their parents (M = 2.75, SD = 0.38) scored significantly higher than those who live in dormitories (M = 2.61, SD = 0.29), rentals (M = 2.61, SD = 0.36), and other types (M = 2.63, SD = 0.34) (Table 2).

### 3.4. Body Mass Index

The BMI results showed that 673 (81.4%) of the AYAs were of normal weight, 88 (10.6%) were underweight, and 66 (8.0%) were overweight (see Table 1). ANOVA test analysis revealed no significant difference between the HPLP-II total score and BMI. However, significant differences were found between BMI and physical activity (F(826) = 9.08, *p* < 0.001), and stress management (F(826) = 3.80, *p* < 0.05) subscales (see Table 2). AYAs with normal BMIs had higher scores in physical activity subscale scores (M = 2.37, SD = 0.61) than underweight (M = 2.16, SD = 0.76) and overweight (M = 2.09, SD = 0.53) students.

### 3.5. University and Academic Year

Post hoc analyses using Tukey’s HSD test were conducted to analyze the significant effect. The results indicated that the participants from the MSUE (M = 2.75, SD = 0.23) scored significantly higher on the HPLP-II than both the NUM (M = 2.51, SD = 0.39) and MUST (M = 2.56, SD = 0.35) universities. In addition, participants from the MSUE scored significantly higher on health responsibility, physical activity, spiritual growth, and stress management subscales (Table 3).

No significant differences between the total HPLP-II scores and the scores on its subscales and students’ academic year, except for the health responsibility subscale (*p* < 0.05).

### 3.6. Location of Graduated School

Table 3 presents the distribution of the HPLP-II scores and subscales according to the location of secondary schools and whether the AYAs graduated in Ulaanbaatar city or rural areas. There was no significant difference between the school location and the HPLP-II total score (t = −1.237; *p* > 0.05). The difference in nutrition scores between the participants from Ulaanbaatar (M = 2.74, SD = 0.42) and rural areas (M = 2.63, SD = 0.31) was statistically significant, with small effect size (Cohen’s *d* = 0.28).

The difference in physical activity scores between the participants from Ulaanbaatar (M = 2.27, SD = 0.65) and rural areas (M = 2.36, SD = 0.61) was statistically significant, with a very small effect size (Cohen’s d = 0.14). The difference in stress management scores between the participants from Ulaanbaatar (M = 2.51, SD = 0.57) and rural areas (M = 2.62, SD = 0.55) was statistically significant, with a small effect size (Cohen’s d = 0.21).

### 3.7. Working While Studying

Of the AYA participants in this study, 382 (46.2%) worked during their studies, while 445 (53.8%) did not. The distributions of the HPLP-II mean scores and subscales according to whether or not the participants worked during their studies are shown in Table 3. The difference in the HPLP-II scores between the participants who work (M = 2.65, SD = 0.34) and not work (M = 2.56, SD = 0.35) was statistically significant, with a small effect size (Cohen’s d = 0.26). Significant differences were found according to the working status of the AYAs and the subscales of health responsibility (t = −3.95, *p* < 0.001), physical activity (t = −5.91; *p* < 0.001), spiritual growth (t = −2.55, *p* < 0.05), and stress management (t = −2.68, *p* < 0.001), with AYAs who worked having higher scores in these areas.

### 3.8. Economic Status

The economic status of the AYAs was categorized into three levels: (1) good (*n* = 109, 13.2%), (2) average (*n* = 629, 76.1%), and (3) less than average (*n* = 89, 10.8%). Post hoc analyses using Tukey’s HSD test were conducted to analyze the significant effect. The results indicated that the participants with higher economic status (M = 2.72, SD = 0.36) scored significantly higher on the HPLP-II than both average (M = 2.61, SD = 0.33) and less than average (M = 2.38, SD = 0.35) groups. Significant differences were found in the total HPLP-II score (F = 25.52, *p* < 0.001) and in the subscale scores among participants with different economic statuses.

### 3.9. Multivariate Test of Gender, Economic Status, and Interaction on HPLP-II

We conducted a MANOVA to examine the effect of gender and economic status on the HPLP-II scores. The findings showed that there was a statistically significant effect for the gender, Wilks’ *λ* = 0.94, F(6816) = 7.85, *p* < 0.001, *η^2^* = 0.05. A significant multivariate main effect was for economic status, Wilks’ *λ* = 0.89, F(121,632) = 7.83, *p* < 0.001, *η^2^* = 0.05. also, the results revealed a significant main effect of economic status of the overall HPLP-II and subscales (*p* < 0.001), with small effect sizes, ranging *η^2^* between 0.02 and 0.06. A significant main effect of gender was found for physical activity (*F*(1821) = 9.91, *p* < 0.01) and interpersonal relationships (F(1821) = 21.29, *p* < 0.001. However, no significance was shown in gender and economic status interaction for the HPLP-II and subscales (*p* > 0.05), indicating that the effect of economic status did not differ between male and female participants (see Table 4).

### 3.10. Health Education Level and Health Subject

Table 5 shows how the overall HPLP-II and subscales scores are distributed according to the AYAs’ health education level and opinion about the ‘Health subject’ taught in their secondary schools. The results showed that 182 (22.0%) of the AYAs had a “good” level of health education, 611 (73.9%) had an “average” level, and 34 (4.1%) had a “poor” level. Significant differences were found in the total HPLP-II score (F = 43.75, *p* < 0.001) and in the subscale scores among participants according to the level of health education. AYAs who rated their health education as “good” had the highest mean score on the HPLP-II (M = 2.74, SD = 0.33). Those who rated their health education as “average” had the second highest mean score (M = 2.58, SD = 0.33), and those who rated their health education as “poor” had the lowest mean score (M = 2.19, SD = 0.42).

Regarding the AYAs’ opinions of the ‘Health subject’ taught in secondary schools, 109 (13.2%) responded “good”, 554 (67.0%) responded “average”, and 160 (19.3%) responded “poor.” A significant difference was found between the AYAs’ opinions on the “Health subject” and the total HPLP-II score (F = 12.86, *p* < 0.001), except for the nutrition subscale. The results showed that the participants who responded positively had the highest HPLP-II scores (M = 2.74, SD = 0.37), followed by the “average” (M = 2.61, SD = 0.32), and the “poor” (M = 2.48, SD = 0.38) responses.

## 4. Discussion

This study examined the health-promoting lifestyle behaviors among Mongolian AYAs in Ulaanbaatar using the Health Promoting Lifestyle Profile-II. Moreover, the study examined the lifestyle behaviors among AYAs with different sociodemographic characteristics from three public universities. The results help us better understand the health behaviors of AYAs and predict their future responsibility for their health and well-being. Our study revealed that the interpersonal relationships and spiritual growth subscale had the highest mean score among the six health-promoting lifestyle subscales. The lowest mean scores were for health responsibility and physical activity. In Mongolia, physical education is a compulsory course during the first year at most universities. In this context, our results indicated that participants in the first and second years were more physically active than those in the third and fourth years, with no statistical significance. These findings suggest that physical education is necessary for AYAs in all years and may positively impact health promotion and the prevention of physical inactivity. The low level of physical activity among AYAs confirms the findings of previous international studies [25,28,30,31,32,33,34,35,36,37,38,39,40]. Access to physical activity is quite challenging in Mongolia, especially on university campuses or areas. It is difficult to exercise outside due to the lack of facilities, and fitness and wellness centers charge high fees. Another potential issue for AYAs is having a busy study schedule with significant home study and assignment requirements.

Previous studies [25,28,30,31,33,34] have also reported gender differences as an important determinant of healthy lifestyles. Studies conducted among university students in Japan, Saudi Arabia, and Kuwait have shown that male students tend to have higher physical activity scores than female students. Additionally, studies have reported that female students are more concerned with health responsibility and nutrition. Similarly, our results showed that male participants tended to score higher than female participants on the physical activity subscale. In Mongolia, physical education is a compulsory course during the first year at most universities. In this context, our results indicated that male participants in the first and second years were more physically active than those in the third and fourth years, though the difference was not statistically significant. This suggests that physical education is necessary for AYAs in all years. It may also positively impact health promotion and the prevention of physical inactivity.

Our results showed that female AYAs had better health-promoting lifestyles, such as health responsibility, nutrition, and interpersonal relationships, than male AYAs. Encouraging health promotion, the concepts of Bandura’s Social Cognitive Theory (SCT) are widely applied [43]. The theory consists of self-efficacy, behavioral capacity, observational learning, reinforcement, and self-control, which dynamically interact among an individual’s behavior, personal and environmental determinants, and how those factors can influence one another [43]. Our study found that female AYAs reported higher health responsibility and stronger interpersonal relationship skills than male AYAs, supporting the predictions of SCT regarding self-efficacy and observational learning. In addition, the findings might indicate that the average female participant scored more than half a standard deviation higher than the average male AYAs, representing a practically meaningful gender gap in the health-promoting behaviors among Mongolian AYAs.

Several studies have reported that students from health colleges tend to have significantly better health-promoting lifestyles than students from non-health colleges, especially with regard to health responsibility and nutrition [25,30,34]. Our study was conducted among AYAs from non-health universities. In Mongolia, most higher education institutions are located in the city [44]. We collected data from the three public universities and their various departments. AYAs from the MSUE had better health-promoting lifestyle practices than those from the NUM and the MUST. They scored significantly higher on the HPLP-II overall and on the health responsibility, physical activity, spiritual growth, and stress management subscales. No significant differences were found among the three groups on the nutrition and interpersonal relationship subscales. Participants from the MSUE had the highest physical activity scores (M = 2.71; SD = 0.36), followed by those from the NUM (M = 2.11; SD = 0.69) and the MUST (M = 2.19; SD = 0.61).

Previous studies have reported differences in AYAs’ HPLP-II scores by the number of academic years spent at universities and colleges. Almutairi et al. [30] conducted a study among Saudi Arabian university students to determine differences between those with health-related backgrounds and those without. They reported significant differences in the physical activity subscale and academic year. However, our findings indicated no significant differences in the total HPLP-II score across academic years, except for the health responsibility subscale. This result was similar to the findings of the study by Al-Kandari et al. [26], who examined the associations between sociodemographic characteristics and the HPLP-II and BMI among Kuwaiti nursing students. Overall, the results of the study suggested that AYAs in the first academic year had slightly higher mean scores in health-promoting lifestyle behaviors compared to upper-year participants. However, the differences were not statistically significant.

Another socio-demographic characteristic associated with health-promoting lifestyles was the place where AYAs lived: with their families, on campus, in a rented apartment, or elsewhere. Our results showed that AYAs who lived with their parents had higher nutrition subscale scores than those who lived apart from their families. This finding is similar to studies in Japan and Iran, which suggest that participants who lived with their families had better eating habits [28,38]. However, our data showed that AYAs who lived separately from their parents tended to have a higher mean score on the stress management subscale than those who lived with their families.

This study found that the differences in health-promoting lifestyles among AYAs were strongly significant according to economic status. We classified the participants’ economic status into three categories: “higher than average,” “average,” and “less than average.” Our data showed that participants with higher or average economic status had significantly higher HPLP-II mean scores than those with lower economic status. Consistent with prior studies among university students in Turkey and Taiwan, our findings confirm that socioeconomic status is a significant determinant of health-promoting lifestyles, as measured by the HPLP-II [25,34]. We conducted a multivariate analysis to determine whether there were gender differences in economic status and HPLP-II. Our findings indicated that economic status is a significant determinant of health-promoting lifestyles, whereas the interaction between gender and economic status showed relatively limited effects. The multivariate analysis showed a statistically significant main effect for gender, Wilks’ λ = 0.94, F(6, 816) = 7.85, * *p* < 0.001, η^2^ = 0.06. According to Cohen [45], this represents a small effect size. The economic status had a significant multivariate main effect, Wilks’ λ = 0.89, F(12, 1632) = 7.83, * *p* < 0.001, η^2^ = 0.05, which also indicates a small effect size. However, the lack of a significant interaction effect (*p* = 0.26) indicates that the relationship between economic status and health behaviors influences similarly for both genders.

The World Health Organization [46] reported that 1.3 billion people worldwide use tobacco products, 80 percent of whom live in low- and middle-income countries. Davaanyam et al. [47] found that age, education level, marital status, and alcohol consumption significantly contribute to the high level of nicotine dependence among Mongolian young adults aged 18 years and older. In our study, 16.9% of the participants smoked cigarettes. Male participants smoked more than female participants. However, the results yielded no statistically significant differences in the HPLP-II total or subscale scores between smokers and non-smokers. Similarly to our findings, the study by Al-Othman et al. [31] suggested that cigarette use was not a significant factor differentiating health-promoting lifestyles among young adults.

Another effective factor in improving health-related behaviors among young adults is educational intervention. Solhi et al. [32] conducted a quasi-experimental control study with two groups of 65 AYAs in an Iranian university. The health education intervention consisted of five training sessions and was delivered to the intervention group. Participants were evaluated using the HPLP-II questionnaire before and after the intervention. Pretest results showed no significant differences between groups and HPLP-II mean scores. After the intervention, however, the HPLP-II mean scores and those of its subscales were significantly higher in the intervention group than in the control group. In Mongolia, Health education was introduced as an independent subject in secondary education in the 2018–2019 school year [48]. To evaluate the curriculum’s effectiveness after secondary school graduation, we categorized AYAs’ opinions into three levels: (1) good, (2) average, and (3) poor. According to our findings, students who had positive opinions about the Health education subject had higher overall HPLP-II scores. In other words, the ‘Health subject’ had a significant effect on students. Our findings suggest that health education for AYAs at an earlier age plays a significant role in promoting health responsibility and well-being.

There are several limitations to this study that should be mentioned. First, this cross-sectional study used a convenience sample from three of the largest public universities in Mongolia. However, this sample may not be representative of Mongolian AYAs. A second limitation concerns the internal consistency of the HPLP-II subscales. While the HPLP-II scale as a whole demonstrated excellent internal consistency (Cronbach’s α = 0.90), the nutrition subscale showed lower reliability (α = 0.52). This finding is consistent with a previous Turkish study that reported similarly low reliability for the nutrition subscale (α = 0.59) [25]. The consistently low values across studies suggest that the nutrition items may not effectively measure the intended construct. This may be due to cultural context or contextual factors, such as dietary pattern disparities or item wording. Nevertheless, the subscale was retained for analysis to ensure comparability with existing studies. Future research could improve reliability by adapting or expanding nutrition-specific items for a similar population. A third limitation is that the questionnaire was self-administered, so the participants’ environment may have influenced their responses. In addition, it is uncertain whether the participants fully comprehended the survey items, as it is possible that time constraints during the final 15–20 min of class caused them to rush and potentially misunderstand while completing the questionnaires, particularly in the items of spiritual growth and health responsibility subscales. Moreover, it is uncertain whether the limited period affected the response rate of the participants. Perhaps, a longer recruitment period would likely reveal a higher response rate and more robust data. Therefore, understanding the health-promoting lifestyle of adolescents and young adults is important for understanding their health choices. Further studies are suggested to examine the replicability of the HPLP-II in broader Mongolian-speaking populations and to include more sociodemographic characteristics. Additionally, the questionnaire did not include information on the personal characteristics, marital status, religious beliefs, ethnicity, and health risk behaviors of university students, all of which are suggested to be investigated in future studies. Furthermore, our study did not include universities from rural areas.

### Recommendations and Implications

Further studies can be drawn from this research. Longitudinal studies to assess the factors of AYAs and health practices are recommended. It would be beneficial to design a structured and culturally appropriate health-promoting lifestyle program among AYAs. For example, create a supportive environment, providing resources and opportunities accessible for promoting physical activity, encouraging healthy diet menus in the university cafeteria with affordable prices, and providing exercise facilities and equipment at university campuses to support physical activity. University is a potential setting to integrate modules on health education and physical activity into broader curriculum requirements. Regarding health responsibility, counseling service programs that are well-equipped with qualified health professionals can improve the AYAs’ personal health problems. These actions require Mongolian health policy makers, university administrators, and public health organizations should involve AYAs’ healthy lifestyles in their plans, and interventional research needs to be proposed to provide healthcare professionals with empirical studies and interventions about healthy lifestyles.

## 5. Conclusions

In conclusion, the results of the current study indicated that AYAs in Mongolian universities lead unhealthy lifestyles and exhibit low levels of physical activity and health responsibility. Significant differences in the HPLP-II scores were found among participants’ economic status, health education level, and opinion of the ‘Health subject.’ AYAs with a good level of health education had higher overall HPLP-II scores and higher scores on each subscale.

There is a need to develop health-promoting practices among AYAs. Our findings examined health-promoting lifestyles and their associated factors. Universities are a potential setting for implementing health-promoting interventions and programs that motivate AYAs to take care of their health during this critical period. These programs could encourage physical activity, a healthy diet, spiritual growth, stress management, and disease prevention. Further studies with a larger sample of AYAs from different contexts and other universities are needed to better examine the impact of sociodemographic background.

## Figures and Tables

**Table 1:** Sociodemographic and healthy lifestyle-related characteristics of the participants.

Variables	Mean (SD)	*n* (%)
Age	19.4 (1.37)	
	Adolescents (age 18–20)	563 (68.07)
	Young adults (age 21–25)	264 (31.93)
Gender		
	Male	330 (39.9)
	Female	497 (60.1)
University		
	MSUE	251 (30.4)
	NUM	277 (33.5)
	MUST	299 (36.2)
Year of study		
	Year 1	308 (37.3)
	Year 2	188 (22.7)
	Year 3	248 (30.0)
	Year 4 and >4	83 (10.0)
Location of secondary school		
	Urban	352 (42.6)
	Rural	475 (57.4)
Type of residence		
	Parental home	383 (46.3)
	Dormitory	168 (20.3)
	Rental	153 (18.5)
	Other	123 (14.9)
Working while studying		
	Yes	382 (46.2)
	No	445 (53.8)
Economic status		
	Good	109 (13.2)
	Average	629 (76.1)
	Less than average	89 (10.8)
BMI		
	<18.5	88 (10.6)
	18.6–24.9	673 (81.4)
	>25.0	66 (8.0)
Cigarette use		
	Yes	140 (16.9)
	No	687 (83.1)
Health education level		
	Good	182 (22.0)
	Average	611 (73.9)
	Poor	34 (4.1)
“Health subject” opinion		
	Good	109 (13.2)
	Average	554 (67.0)
	Poor	160 (19.3)
	N/A	4 (0.5)

**Table 2:** Means, standard deviations, gender, residential type, and BMI differences for the variables.

Variables (*n*)	HR	N	PA	SG	IR	SM	HPLP-II
	M (SD)	M (SD)	M (SD)	M (SD)	M (SD)	M (SD)	M (SD)
Gender							
Men (330)	2.24 (0.50)	2.65 (0.40)	2.47 (0.61)	2.78 (0.55)	2.77 (0.52)	2.59 (0.54)	2.59 (0.35)
Women (497)	2.35 (0.47)	2.70 (0.33)	2.23 (0.63)	2.76 (0.55)	2.96 (0.51)	2.56 (0.58)	2.61 (0.35)
Total HPLP-II	2.31 (0.49)	2.68 (0.36)	2.32 (0.63)	2.77 (0.55)	2.89 (0.52)	2.57 (0.56)	2.60 (0.35)
t value	−3.11 **	−2.19 *	5.39 ***	0.33	−4.80 ***	0.83	−0.52
Cohen’s *d*	0.25	0.13	−0.38	−0.04	0.37	0.05	0.06
Residential status							
Parental home (383)	2.27 (0.50)	2.75 (0.38)	2.27 (0.64)	2.75 (0.59)	2.91 (0.54)	2.51 (0.58)	2.59 (0.34)
Dormitory (168)	2.34 (0.44)	2.61 (0.29)	2.41 (0.60)	2.79 (0.49)	2.87 (0.42)	2.66 (0.49)	2.62 (0.33)
Rental (153)	2.34 (0.47)	2.61 (0.36)	2.38 (0.62)	2.80 (0.52)	2.83 (0.55)	2.63 (0.56)	2.61 (0.35)
Other (123)	2.31 (0.51)	2.63 (0.34)	2.29 (0.67)	2.74 (0.54)	2.94 (0.54)	2.60 (0.59)	2.60 (0.38)
F value	1.29	9.64 ***	2.60	0.40	1.39	3.52 *	0.34
BMI							
<18.4 (88)	2.35 (0.54)	2.73 (0.34)	2.16 (0.76)	2.66 (0.64)	2.87 (0.57)	2.47 (0.61)	2.54 (0.39)
18.5–24.9 (673)	2.31 (0.48)	2.68 (0.35)	2.37 (0.61)	2.78 (0.54)	2.88 (0.51)	2.60 (0.56)	2.60 (0.34)
>25.0 (66)	2.19 (0.46)	2.64 (0.43)	2.09 (0.53)	2.77 (0.55)	3.03 (0.53)	2.44 (0.53)	2.53 (0.33)
F value	2.49	1.31	9.08 ***	1.96	2.75	3.80 *	2.07

Note: * *p* < 0.05, ** *p* < 0.01, *** *p* < 0.001. HR—Health responsibility; N—nutrition; PA—physical activity; SG—spiritual growth; IR—interpersonal relationship; SM—stress management.

**Table 3:** HPLP-II scores according to the university, the location of the graduate school, and the working status.

Variables (*n*)	HR	N	PA	SG	IR	SM	HPLP-II
	M (SD)	M (SD)	M (SD)	M (SD)	M (SD)	M (SD)	M (SD)
University							
MSUE (251)	2.54 (0.30)	2.66 (0.24)	2.71 (0.36)	2.85 (0.34)	2.86 (0.33)	2.84 (0.37)	2.75 (0.23)
NUM (277)	2.15 (0.50)	2.69 (0.40)	2.11 (0.70)	2.68 (0.63)	2.87 (0.60)	2.45 (0.64)	2.51 (0.39)
MUST (299)	2.26 (0.53)	2.69 (0.39)	2.20 (0.61)	2.78 (0.60)	2.93 (0.58)	2.45 (0.54)	2.56 (0.35)
*F* value	47.73 ***	0.46	83.15 ***	5.96 **	1.44	46.65 ***	36.13 ***
Location of the school of graduation							
Ulaanbaatar (352)	2.27 (0.51)	2.74 (0.42)	2.27 (0.65)	2.73 (0.60)	2.91 (0.54)	2.51 (0.57)	2.58 (0.36)
Rural (475)	2.33 (0.47)	2.63 (0.31)	2.36 (0.61)	2.79 (0.51)	2.88 (0.50)	2.62 (0.55)	2.60 (0.34)
*t* value	−1.81	4.04 ***	−2.02 *	−1.59	0.76	−2.98 **	−1.24
Cohen’s *d*	−0.13	0.28	−0.14	−0.11	0.05	−0.21	−0.09
Working status							
No (445)	2.25 (0.49)	2.70 (0.38)	2.20 (0.65)	2.72 (0.59)	2.89 (0.55)	2.52 (0.60)	2.56 (0.35)
Yes (382)	2.38 (0.47)	2.65 (0.33)	2.46 (0.58)	2.82 (0.50)	2.90 (0.50)	2.63 (0.51)	2.65 (0.34)
*t* value	3.95 ***	1.95	−5.91 ***	2.55 *	−0.18	−2.68 **	−3.61 ***
Cohen’s *d*	−0.27	0.14	−0.42	−0.18	0.02	−0.20	−0.26
Economic status							
Good (109)	2.34 (0.61)	2.83 (0.37)	2.34 (0.73)	2.91 (0.63)	3.11 (0.62)	2.68 (0.59)	2.72 (0.36)
Average (629)	2.34 (0.45)	2.68 (0.33)	2.36 (0.61)	2.77 (0.52)	2.87 (0.48)	2.60 (0.53)	2.61 (0.33)
Less than average (89)	2.04 (0.47)	2.52 (0.44)	2.06 (0.62)	2.56 (0.63)	2.77 (0.59)	2.23 (0.61)	2.38 (0.35)
*F* value	15.75 ***	18.82 ***	8.84 ***	9.92 ***	12.43 ***	19.85 ***	25.52 ***

Note: * *p* < 0.05, ** *p* < 0.01, *** *p* < 0.001. HR—Health responsibility; N—nutrition; PA—physical activity; SG—spiritual growth; IR—interpersonal relationship; SM—stress management.

**Table 4:** MANOVA results for gender, economic status, and their interaction on HPLP-II.

Variables	Wilk’s λ	F	df	*p*	η^2^
Gender	0.94	7.85	6816	<0.001 **	0.055
Economic status	0.89	7.83	121,632	<0.001 **	0.054
Gender and economic status	0.98	1.22	121,632	0.26	0.01
Health responsibility					
Gender		2.02	1821	0.16	0.002
Financial status		14.26	2821	<0.001 **	0.034
Gender and economic status		0.72	2821	0.49	0.002
Nutrition					
Gender		1.78	1821	0.18	0.002
Financial status		17.75	2821	<0.001 **	0.041
Gender and financial status		1.28	2821	0.28	0.003
Physical activity					
Gender		9.91	1821	0.002 *	0.012
Financial status		11.38	2821	<0.001 **	0.027
Gender and economic status		1.44	2821	0.24	0.003
Spiritual growth					
Gender		0.005	1821	0.94	0.000
Financial status		9.32	2821	<0.001 **	0.022
Gender and economic status		0.41	2821	0.66	0.001
Interpersonal relationships					
Gender		21.30	1821	<0.001 **	0.025
Financial status		8.79	2821	<0.001 **	0.021
Gender and economic status		2.60	2821	0.08	0.006
Stress management					
Gender		1.59	1821	0.21	0.002
Financial status		20.67	2821	<0.001 **	0.050
Gender and economic status		0.08	2821	0.92	0.000
HPLP-II					
Gender		0.34	1821	0.56	0.000
Financial status		24.50	2821	<0.001 **	0.056
Gender and economic status		1.24	2821	0.29	0.003

Note: * *p* < 0.01, ** *p* < 0.001.

**Table 5:** Distribution of the HPLP-II scores according to the health education level and opinion of the Health subject.

Variables (*n*)	HR	N	PA	SG	IR	SM	HPLP-II
	M (SD)	M (SD)	M (SD)	M (SD)	M (SD)	M (SD)	M (SD)
HE level							
Good (182)	2.40 (0.55)	2.81 (0.40)	2.42 (0.68)	2.98 (0.55)	3.11 (0.52)	2.65 (0.63)	2.74 (0.33)
Average (611)	2.31 (0.45)	2.66 (0.32)	2.33 (0.60)	2.72 (0.52)	2.85 (0.48)	2.57 (0.53)	2.58 (0.33)
Poor (34)	1.78 (0.56)	2.36 (0.54)	1.72 (0.59)	2.43 (0.81)	2.50 (0.73)	2.24 (0.70)	2.19 (0.42)
F (2)	23.97 **	28.33 **	18.17 **	23.52 **	28.97 **	7.91 **	43.75 **
HE subject opinion							
Good (109)	2.42 (0.63)	2.76 (0.41)	2.42 (0.64)	2.97 (0.58)	3.07 (0.62)	2.72 (0.61)	2.74 (0.37)
Average (554)	2.34 (0.42)	2.66 (0.33)	2.37 (0.60)	2.75 (0.49)	2.87 (0.48)	2.62 (0.52)	2.61 (0.32)
Poor (160)	2.12 (0.55)	2.69 (0.42)	2.10 (0.67)	2.69 (0.67)	2.86 (0.55)	2.31 (0.62)	2.48 (0.38)
Do not know (4)	2.31 (0.49)	2.68 (0.36)	2.32 (0.63)	2.77 (0.55)	2.89 (0.52)	2.57 (0.56)	2.60 (0.35)
F (3)	10.91 **	2.38	9.17 **	6.20 **	4.71 *	16.15 **	12.86 **

Note: HR-Health responsibility, N-nutrition, PA-physical activity, SG-spiritual growth, IR-interpersonal relationship, SM-stress management. * *p* < 0.05, ** *p* < 0.001.

## Data Availability

The data presented in the study are available on request from the corresponding author. The data are not publicly available due to privacy reasons.

## Abbreviations

The following abbreviations are used in this manuscript:

AYA	Adolescents and young adults
CFI	Comparative Fit Indices
CFA	Confirmatory Factor Analysis
EFA	Exploratory Factor Analysis
GFI	Goodness of Fit Indices
BMI	Body Mass Index
HR	Health Responsibility
IR	Interpersonal Relationship
IRB	Institutional Review Board
MSUE	Mongolian State University of Education
MUST	Mongolian University of Technology and Science
N	Nutrition
NCD	Noncommunicable disease
NUM	National University of Mongolia
HPLP-II	Health-Promoting Lifestyle Profile II
RMSEA	Root Mean Square Error of Approximation
SCT	Social Cognitive Theory
SG	Spiritual Growth
SM	Stress Management
SPSS	Statistical Package for the Social Sciences
TLI	Tucker and Lewis Index
PA	Physical Activity
WHO	World Health Organization
